# Authorship then and now

**DOI:** 10.7554/eLife.76242

**Published:** 2022-01-18

**Authors:** Eve Marder

**Affiliations:** 1 Volen Center and Biology Department, Brandeis University Waltham United States

**Keywords:** Living Science, authorship, Guido Guidotti

## Abstract

A researcher should only be an author on a paper if they have contributed to it in a substantive way.

I recently read Nancy Kleckner’s appreciation of her late husband, Guido Guidotti, who died in August 2021 after a long and distinguished career at Harvard University (Kleckner, 2021). I strongly recommend Kleckner’s article – which is complemented by contributions from a large number of Guidotti ’s colleagues and friends – whether or not you knew him and his work on the biochemistry and biophysics of a range of fundamental proteins and processes. His work illustrates brilliantly how previous generations of biochemists and biophysicists made discoveries without the benefits (and perhaps curses) of molecular techniques. Instead, they spent hours in cold rooms, and used brute force, cleverness, and hard work to purify proteins and characterize their properties and functions.

Guidotti also had interesting ideas on the authorship of scientific papers. Back in 1960, when he published his first paper, only those researchers who had had a significant role in generating the data were listed as an author, and it was not uncommon for the papers from PhD theses to have just one author because a PhD thesis was meant to be an independent piece of work. The community of scientists in any field of research was so small back then that people in the field would likely know that a first-time author was a PhD student in an established scientist’s laboratory.

In accord with this policy, my own PhD papers were single-authored, as were those of several of my lab colleagues. At first our thesis supervisor – a superb electrophysiologist called Allen Selverston – only signed papers from his lab when he had actually participated in collecting the data. However, shortly after I completed and published my thesis papers in the mid-1970s, it became almost unheard of to have single-authored papers from students and postdocs. So, in electrophysiology, as in other areas of biology, it became customary for lab heads to be the last author on papers from their lab. This progression has also been described explicitly for the field of meiosis (Zickler, 2020).

**Figure 1:**
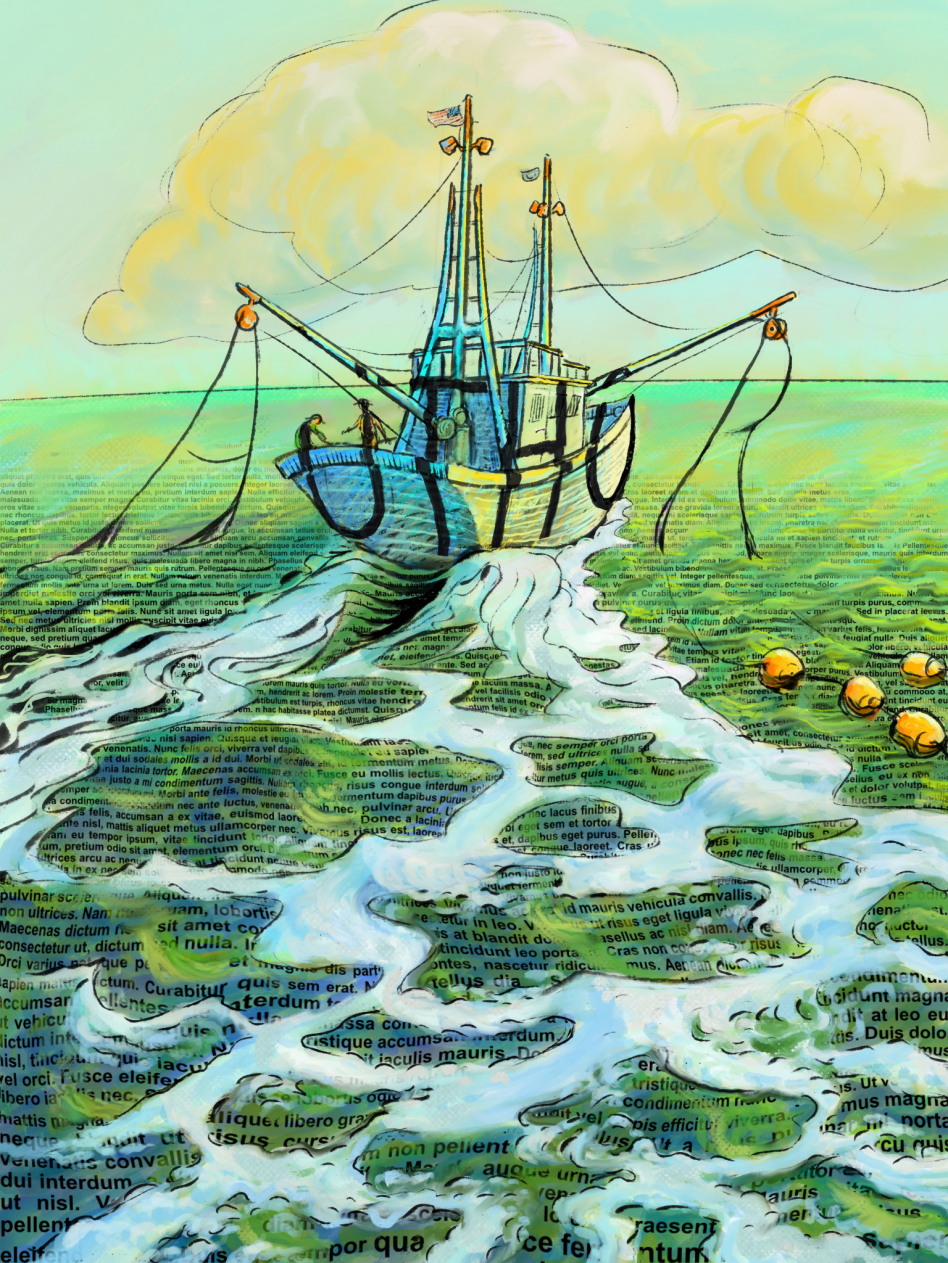
Views on who should be included as an author on a scientific paper have changed over time.

Illustration: Ben Marder.

However, Guidotti maintained his policy of only putting his name on a paper if he had done some amount of bench work to collect the data as he felt that this was the right thing to do, and he adhered to this policy long after it was no longer in his interest to do so. Indeed, according to a profile of Guidotti that was published in the newsletter of the American Society for Cell Biology (ASCB) in 2003, he only abandoned this policy when it started to cause problems with funding agencies: "Guidotti says his days as an unacknowledged collaborator ended abruptly in 1985 when he was called on the carpet before an NIH study section. 'They said I was plagiarizing by using these articles that were published without my name on them,' Guidotti recalls. 'So now I put my name on all the papers, even the ones I didn’t work on.'" (The ASCB profile of Guidotti is included as an appendix in Kleckner, 2021). Nonetheless, he continued to resist having his name on papers from colleagues and former students to which he made "only" intellectual contributions. After his death, a number of former students asked that such "unacknowledged" contributions be listed in the CV provided in Kleckner’s article.

Similarly, I have refrained from signing papers on which I had literally no input. For example, when one of my postdocs wrote a paper on capacitance in cables (Taylor, 2012), I declined to be an author because, as I told him, I couldn’t contribute to the paper! But recently, I had an experience similar to Guidotti’s. I shared a grant with another investigator, and I didn’t put my name on papers that were funded by the grant but on which I had no specific role in the actual work or writing. Although these papers appropriately cited the grant that had paid for the work, there was a reviewer on the renewal application who refused to acknowledge that those papers represented progress on the grant’s objectives because my name wasn’t included on them. I was being punished for not taking credit for work to which I had only minimally contributed. How could this have happened as recently as 2019, at a time when journals are being more specific about author contributions in papers, and institutions require researchers to attend courses on responsible conduct in science that often include discussions about authorship?

Of course, the world has gone awry in numerous ways, many of them far more important than the question of who signs the papers from our labs. Meanwhile, some of the old ways are best: we should only be authors on papers when we have contributed in a substantive way. That should go without saying, but as with so much in today’s world, ethical principles seem increasingly at odds with practice.

## Note

This essay is part of the Living Science collection.
